# Enhancing readiness for exposure and response prevention in obsessive compulsive disorder: ketogenic metabolic therapy as a candidate readiness adjunct

**DOI:** 10.3389/fpsyg.2026.1770306

**Published:** 2026-09-15

**Authors:** Nicole Laurent

**Affiliations:** Independent Researcher, Vancouver, WA, United States

**Keywords:** clinical staging, ERP, exposure and response prevention, KD, ketogenic diet, ketogenic metabolic therapy, KMT, metabolic psychiatry

## Abstract

Exposure and response prevention (ERP) remains a first-line psychological treatment for obsessive compulsive disorder (OCD), yet a clinically meaningful subset of patients cannot initiate, tolerate, or complete an adequate ERP dose even after step up in format, frequency, pharmacotherapy augmentation, or level of care. These failures are often interpreted as noncompliance, but they more often reflect a persistent capacity gap that limits between-session exposure practice and response prevention. This conceptual analysis positions ketogenic metabolic therapy (KMT) as a candidate adjunct when low capacity prevents delivery of an adequate ERP trial or persists after nonresponse. OCD evidence for KMT remains limited to case level reports that include standardized symptom measures and describe remission during nutritional ketosis, including one report in which KMT preceded successful ERP completion. These observations support a narrower clinical question that centers on capacity restoration rather than direct symptom treatment. ERP readiness is conceptualized across clinical, functional, and psychosocial domains that shape distress tolerance, cognitive efficiency, daily feasibility, and accommodation. Measurement-based care anchors decision points using adherence to between session exposure practice, completion of an adequate ERP dose, and established response and remission thresholds on validated symptom and global severity measures. Stepped care and staging checkpoints define pragmatic windows to test whether KMT increases ERP engagement, reduces refusal and drop out, and improves symptom severity and functioning beyond usual escalation alone.

## 1 Introduction

Exposure and response prevention (ERP) is a first line psychological treatment for obsessive compulsive disorder (OCD) and is recommended in clinical practice guidelines (Koran et al., 2007; Janardhan Reddy et al., 2017). Stepped care protocols specify escalation in ERP intensity and format when response is insufficient, while clinical staging models add a temporal and prognostic dimension that supports stage matched monitoring and management over time (Gilliam et al., 2010; Tolin et al., 2011; Fontenelle and Yücel, 2019; Benatti et al., 2022; Moreira-de-Oliveira et al., 2025). Greater clinical complexity in OCD often includes higher comorbidity burden and poorer insight, both of which complicate treatment planning and have been repeatedly characterized in the OCD literature (Lochner et al., 2014; de Avila et al., 2019; Broekhuizen et al., 2023; Wolf et al., 2023).

Guidelines and reviews emphasize that ERP outcomes depend on adherence to prescribed exposure and response prevention procedures, and a clinically meaningful proportion of patients discontinue or show limited improvement (Aderka et al., 2011; Hezel and Simpson, 2019; Leeuwerik et al., 2019; Ojalehto et al., 2020). Practical barriers further contribute to underutilization of evidence based care, including limited availability, time and distance constraints, and financial obstacles (Hathorn et al., 2021; Senter et al., 2021; Frank et al., 2023; Bey et al., 2025; Milgram et al., 2025).

Stepped care models commonly begin with lower intensity, technology delivered or guided self help CBT ERP options that can function as an initial step before a more intensive face to face ERP course, with remotely delivered CBT positioned as a scalable access pathway and as part of stepped care service delivery (Hoppen et al., 2021; Salazar de Pablo et al., 2023; Polak and Tanzer, 2024). Pharmacotherapy evidence supports the role of Selective-Serotonin Reuptake Inhibitors (SSRIs) and clomipramine as established OCD treatments that are commonly paired with psychological care when symptom burden or response warrants combined approaches (Janardhan Reddy et al., 2017; Cohen et al., 2024). Intensive outpatient, residential, and inpatient formats are typically used for more severe, complex, or refractory presentations, with evidence indicating large symptom reductions from admission to discharge that are often maintained at follow up (Grøtte et al., 2018; Zisler et al., 2024; Mogan et al., 2025).

Readiness failures are commonly misread as noncompliance, yet they often reflect a capacity gap that persists even after appropriate ERP sequencing and escalation (Hezel and Simpson, 2019; Leeuwerik et al., 2019). In this conceptual analysis, ketogenic metabolic therapy (KMT) is positioned as a candidate adjunct to test at stepped care and staging checkpoints when low readiness prevents delivery of an adequate ERP trial or persists after nonresponse, with success defined by improved ERP engagement and completion alongside symptom severity and functioning.

This conceptual analysis evaluates and synthesizes evidence at the intersection of evidence-based psychotherapy delivery, implementation and engagement science, measurement-based care, and metabolic psychiatry. The central problem is that a clinically meaningful subset of patients report being unable to initiate, tolerate, or complete an adequate ERP dose even after escalation, with the limiting mechanism framed as a persistent capacity gap that constrains between session exposure practice and response prevention. The evidence for the use of KMT in OCD is currently limited and case-based yet describes changes during nutritional ketosis in domains that plausibly intersect with ERP readiness, making a delivery-focused, endpoint anchored model necessary to generate testable clinical questions relevant to ERP. The model logic specifies readiness limiting barriers, maps them to candidate KMT associated outcome domains, and anchors evaluation to stepped care and staging decision points using ERP participation endpoints alongside symptom severity and functioning.

Evidence was acquired through integrative narrative review and conceptual synthesis across two bodies of literature. The first addresses ERP readiness limiting barriers and clinical decision making across stepped levels of care and staging checkpoints, including measurement anchors for participation and adequacy of ERP dose. The second addresses published KMT clinical evidence relevant to psychiatric, functional, and psychosocial domains that commonly constrain ERP engagement. These literatures were integrated through conceptual mapping of readiness limiting barriers to candidate KMT associated outcome domains, with evaluation criteria defined in psychotherapy delivery terms: ERP initiation, completion, and between session exposure follow through, alongside symptom severity and functioning.

## 2 Positioning KMT within ERP readiness frameworks

KMT may eventually be shown to have broader psychiatric relevance, yet the clinically actionable question in OCD care is whether it restores capacity for ERP when standard sequencing and escalation have not. This conceptual analysis examines KMT through an ERP readiness lens, specifying stepped care and staging checkpoints and defining success in terms of ERP engagement and completion. Ketogenic metabolic therapy (KMT) refers to clinician supervised ketogenic dietary therapies that induce nutritional ketosis through carbohydrate restriction, increased dietary fat intake, and structured protocols for education, supplementation, monitoring, and discontinuation (Kossoff et al., 2018). In neurology, ketogenic dietary therapies are established nonpharmacologic treatments for medication refractory epilepsy, supported by consensus recommendations that standardize patient selection and clinical management (Kossoff et al., 2018; Klepper et al., 2020; Martin-McGill et al., 2020; Kolic et al., 2021).

Psychiatric applications of KMT build on the neurological precedent that nutritional ketosis can alter brain energy metabolism and are increasingly considered within a transdiagnostic framework focused on biological processes shared across neuropsychiatric disorders (Norwitz et al., 2020; Sethi and Ford, 2022; Anderson et al., 2024; Laurent, 2024; Bellamy and Laurent, 2025; Sethi et al., 2026). Nutritional ketosis provides ketones as an alternative cerebral fuel and is proposed to support mitochondrial function and reduce oxidative and inflammatory stress (Norwitz et al., 2020; Lounici et al., 2024; Dawson et al., 2025; Sethi et al., 2026). Across syntheses the clinical literature remains early and methodologically heterogeneous, with recurring limitations that include variable dietary formulations, incomplete reporting of carbohydrate targets, and inconsistent verification of ketosis (Dietch et al., 2023; Janssen-Aguilar et al., 2026).

One systematic review and meta-analysis focused on depression and anxiety symptoms found modest improvements in depressive symptoms with stronger effects in studies that monitored ketones and inconclusive findings for anxiety, supporting the need for standardized protocols, fidelity measures, and prespecified outcomes in future trials (Janssen-Aguilar et al., 2026). Metabolic factors implicated in OCD have been described alongside a mechanistic rationale for ketogenic metabolic therapy, while emphasizing that prospective OCD dietary trials are needed to determine whether KMT can improve symptoms (Lounici et al., 2024; Dawson et al., 2025).

OCD-specific evidence remains limited to multiple peer-reviewed case-level publications reporting remission of symptoms. Published OCD case reports and case series include standardized symptom outcomes during KMT (Table 1). A case series of three individuals who initiated KMT for treatment-resistant OCD in the hopes of alleviating symptoms underwent interviews and completed initial and follow-up Yale-Brown Obsessive Compulsive Scale (Y-BOCS) assessments showing scores of 4, 3 and 0, respectively. Two of the patients reported significant improvements and remission within 2 weeks, with the third reporting complete cessation of symptoms after 1 year. All three were reported to maintain improvements over extended periods, with deviations from the diet leading to symptom recurrence (MacDonald and Palmer, 2025).

**Table 1:** Case report OCD outcomes and ERP readiness indicators during KMT.

Case report or series	Description	Measurement and timeline	Key findings relevant to ERP readiness
MacDonald and Palmer (2025)	Three patients with treatment-resistant OCD using KMT.	Y-BOCS decreased by an average of 21 points (≈ 90.5% reduction). Individual cases included 27 → 4; 22 → 3; 21 → 0.	Rapid and durable symptom improvement; functional return; relapse on discontinuation.
Calabrese (2025)	37-year-old woman with OCD + ulcerative colitis.	Y-BOCS 12 → 0 within 12 weeks with CGI-S = 1; corroborated by FOCI = 0 and PHQ-9 = 0.	Full OCD remission and affective stabilization.
Laurent and Tague (2025)	26-year-old man with treatment-resistant OCD who initiated KMT before ERP.	DOCS Symmetry 12 → 7 → 5 → 2, DASS-21 Total 32 → 6 → 4 → 2 over 95 weeks, with the final scores recorded at the 95-week extended follow-up.	Early symptom decline before ERP, sustained remission for nearly 2 years at follow-up, improved emotional regulation aiding ERP completion.
Calabrese et al. (2024)	Case 1, adult man with comorbid OCD in depression and anxiety case series.	PHQ-9 17 at initiation, remitted within 5 weeks with PHQ-9 ≤ 4 | GAD-7 16 at initiation, 16 → 8 within 1 week, score of 0 at 6 weeks. Y-BOCS baseline not reported, Y-BOCS 1 at 12 weeks, SCS 3 → 4.6 over 4 weeks.	KMT was associated with reduced obsessive compulsive symptoms during the intervention, supporting a hypothesis that symptom reduction may improve capacity to initiate and sustain ERP tasks when symptoms previously limited engagement.

Table summarizes case level OCD outcomes and readiness relevant indicators reported during KMT. KMT, Ketogenic metabolic therapy; ERP, Exposure and response prevention; Y-BOCS, Yale Brown Obsessive Compulsive Scale; DOCS, Dimensional Obsessive Compulsive Scale; DASS-21, Depression Anxiety Stress Scales 21 item; PHQ-9, Patient Health Questionnaire 9 item; GAD-7, Generalized Anxiety Disorder 7 item; FOCI, Florida Obsessive Compulsive Inventory; CGI-S, Clinical Global Impressions Severity; SCS, Self-Compassion Scale.

Another case report presented a 37-year-old woman with OCD and ulcerative colitis who achieved complete remission of OCD during KMT, with Y-BOCS decreasing to 0 from 12 (Calabrese, 2025). Another case report within a depression and anxiety case series described a 32-year-old man with a history of comorbid OCD who reported reduced obsessive thoughts, with Y-BOCS reported as 1 at 12 weeks and no baseline Y-BOCS available (Calabrese et al., 2024).

Relevant to KMT and ERP specifically, a case report exists describing how a 26-year-old man with treatment-resistant OCD utilized KMT prior to seeking ERP therapy. Of interest is the reported subjective experience of the patient that KMT’s significant positive mood effects enhanced his ability to complete ERP treatment. Long-term outcomes were evaluated over a 95-week follow-up, during which the patient achieved and sustained remission of OCD symptoms, along with improvements in mood, emotional regulation, and quality of life (Laurent and Tague, 2025).

These uncontrolled reports require cautious interpretation given the potential for researcher allegiance bias and the absence of independent replication, and they do not justify broad conclusions about KMT as OCD treatment. They do, however, justify a narrower, testable question of whether KMT could improve readiness relevant capacities that determine whether ERP can be initiated, tolerated, and completed. To ground that question in clinical practice, the next section defines ERP readiness as a dynamic construct with identifiable domains that can be evaluated in routine care.

## 3 Clinical, functional, and psychosocial indicators that guide treatment decisions

Readiness for ERP is evaluated in whether prescribed exposure practice and response prevention occur consistently between sessions, not only in session attendance (Hezel and Simpson, 2019; Ojalehto et al., 2020; Tjelle et al., 2021). Readiness indicators map onto three domains that repeatedly predict adherence and outcome, clinical factors that shape distress tolerance and task completion during exposures, functional factors that constrain whether exposure work can fit into daily life, and psychosocial factors that reduce or amplify accommodation and avoidance (Craske et al., 2014; Leeuwerik et al., 2019). Family accommodation shows consistent association with obsessive compulsive symptom severity and decreases following individual and family focused cognitive behavioral therapy, supporting its use as a readiness domain that can be targeted alongside exposure work (Thompson-Hollands et al., 2015; Wu et al., 2016; Hamatani et al., 2020; Hermida-Barros et al., 2024).

Clinical indicators include symptom severity, acuity, comorbidity, insight, and treatment history (Janardhan Reddy et al., 2017). Symptom severity is commonly operationalized with the Yale-Brown Obsessive Compulsive Scale and by estimating daily time spent in obsessions and compulsions (Goodman et al., 1989; Janardhan Reddy et al., 2017). Mild and moderate presentations are typically managed in outpatient care, while severe and extreme presentations more often require greater structure and frequency, particularly when rituals occupy many hours per day and functioning is substantially constrained (Janardhan Reddy et al., 2017; Grøtte et al., 2018). Comorbid severe depression, mania, and psychosis complicate readiness and level-of-care decisions, particularly when suicidality or significant medical risk is present (Janardhan Reddy et al., 2017; de Avila et al., 2019). Poor insight is associated with greater symptom burden and lower quality of life and tends to reduce response to both medication and behavioral treatments (Hezel and Simpson, 2019; Wolf et al., 2023). A history of multiple adequate medication or ERP trials without sufficient benefit raises the question of whether alternative or adjunctive strategies are needed before asking the person to attempt another course of exposures (Janardhan Reddy et al., 2017; Hezel and Simpson, 2019; Fineberg et al., 2020; Mao et al., 2022).

Meta analyses and reviews identify neurocognitive vulnerability in OCD, with reduced processing speed proposed as one contributor to broader underperformance (Shin et al., 2014; Snyder et al., 2015; Abramovitch et al., 2019; Kashyap and Abramovitch, 2021). Symptom severity shows a small to moderate association with cognitive performance (Abramovitch et al., 2019). Co-occurring depressive symptoms have also been associated with worse cognitive performance in obsessive compulsive disorder (Mondal and Kumar, 2020). Consistent with this readiness framing, diminished working memory has been identified as a predictor of poorer response to ERP based cognitive behavioral therapy (Hamatani et al., 2020).

Functional indicators capture the impact of OCD on work or school, self-care, and independent living (Janardhan Reddy et al., 2017; Mataix-Cols et al., 2022). Patients who are still attending school or work, though with reduced productivity or increased distress, often can engage in standard outpatient ERP if schedules are adjusted and employers or educators are informed (Janardhan Reddy et al., 2017). Those who have stopped working or attending school, who cannot leave home without extensive rituals, or who rely heavily on family for basic tasks are more likely to benefit from intensive outpatient, partial hospital, or residential settings where exposure work can be embedded into a structured day rather than added to an already overwhelmed routine (Janardhan Reddy et al., 2017; Grøtte et al., 2018; Zisler et al., 2024; Mogan et al., 2025).

Psychosocial indicators include social support, family accommodation, interpersonal conflict, and environmental stability (Leeuwerik et al., 2019). High levels of accommodation, such as family members participating in rituals, answering reassurance questions repeatedly, or restructuring the household to minimize triggers, are consistently associated with worse outcomes and lower readiness for ERP (Wu et al., 2016; Hermida-Barros et al., 2024). Social isolation, relationship strain, and limited financial or practical resources further reduce the likelihood that ERP will succeed without additional support (Leeuwerik et al., 2019). Conversely, families who are willing to learn about OCD and reduce accommodation can become powerful allies in exposure work (Thompson-Hollands et al., 2015; Hermida-Barros et al., 2024).

For the purposes of this conceptual analysis, these three domains can be used to identify moments when standard step-up strategies have been exhausted, and the patient remains in a state of low readiness despite best efforts. Those moments may be where experimental integration of metabolic interventions is most ethically and clinically justifiable if safety can be assured.

Measurement-based care offers a practical way to define adequate trials and to decide when to change course. Symptom scales such as the Y-BOCS and functioning measures such as work or school participation and quality of life indices can be used together to determine whether a given level of care and ERP dose has produced meaningful change. Validated ERP readiness instruments do not exist as a single, field standard construct that maps cleanly onto the domains of clinical factors, functional factors, and psychosocial factors (Simpson et al., 2010; Cole Monaghan et al., 2015). OCD literature measures readiness indirectly through validated instruments that each capture one readiness relevant domain, most often adherence, motivation, expectancy or credibility, and accommodation (Simpson et al., 2010; Cole Monaghan et al., 2015; Berg et al., 2025).

Readiness can be tracked using severity, adherence, and response thresholds that already guide ERP decision making. Clinically, poor response has been associated with lack of adherence, poor insight, comorbid depression, and higher OCD severity, with severity assessed on the Y-BOCS (Hezel and Simpson, 2019). Functional change can be anchored to operational response and remission criteria that capture meaningful improvement in clinician rated functioning and quality of life, with response defined by Y-BOCS change plus Clinical Global Impressions Improvement (CGI-I) and remission defined by Y-BOCS plus Clinical Global Impressions Severity (CGI-S) (Mataix-Cols et al., 2022). Between session exposure completion can be monitored as an adherence marker because low adherence to completing exposures assigned between sessions has predicted higher post-treatment symptom severity (Hezel and Simpson, 2019). Psychosocially, accommodation patterns remain a practical readiness target because they can maintain rituals and undermine response prevention in daily life (Thompson-Hollands et al., 2015; Wu et al., 2016; Hermida-Barros et al., 2024). Evaluated together, these indicators can specify when ERP sequencing has been adequate and when a decision point has been reached, thereby framing whether a candidate adjunct can restore readiness for ERP.

## 4 Readiness failures across levels of care and KMT as a candidate readiness adjunct

Readiness constraints appear across levels of care, and stepped care models specify escalation in ERP intensity and format. Stepped care protocols have tested low intensity, self-directed or internet delivered ERP based steps with step up to therapist delivered ERP when response is insufficient, and clinical guidelines describe outpatient care for mild to moderate illness and inpatient care for severe, treatment resistant, or safety complicated presentations (Gilliam et al., 2010; Tolin et al., 2011; Janardhan Reddy et al., 2017; Aspvall et al., 2021). Low intensity and standard outpatient delivery depend on patients practicing exposures and response prevention between sessions, and lower adherence to completing assigned exposures has predicted higher post treatment symptom severity (Hezel and Simpson, 2019; Ojalehto et al., 2020; Tjelle et al., 2021). Higher intensity programs, including intensive outpatient and inpatient formats, have been evaluated in OCD and have shown considerable symptom reduction that is often maintained after discharge (Grøtte et al., 2018; Zisler et al., 2024; Mogan et al., 2025).

Ketogenic metabolic therapy is proposed as a readiness adjunct for situations where ERP engagement and completion have remained out of reach despite adequate sequencing and escalation. Ketogenic metabolic therapy involves nutritional strategies that shift the body’s primary fuel source toward ketone-based metabolism. Implementation introduces practical barriers that can affect adherence without structured guidance and multidisciplinary support (Laurent et al., 2024). In neurology, ketogenic interventions have a long history in the management of refractory epilepsy and are being explored for a range of neuropsychiatric conditions. From the perspective of ERP readiness, the question is not whether KMT “treats OCD” in a direct sense but whether it could improve domains that limit engagement, such as depressive symptoms including fatigue, and cognitive inefficiency (Snyder et al., 2015; Mondal and Kumar, 2020; Kashyap and Abramovitch, 2021).

A readiness-oriented view suggests at least three hypothetical pathways. First, if KMT were to reduce overall symptom burden or dampen the intensity of obsession related distress, even without full remission, exposures might become more tolerable and less likely to trigger shutdown or drop out. Second, if KMT were to improve daytime alertness, executive functioning, or cognitive control in some individuals, it could make it easier to plan, initiate, and sustain exposure tasks. Third, if KMT were to stabilize mood or reduce comorbid depressive symptoms for some patients, motivation and hope might increase enough to re-engage with ERP that previously felt impossible.

## 5 Decision points and research questions for integrating KMT with ERP

If KMT is studied as a readiness adjunct rather than a stand-alone alternative to ERP, decision points should be anchored to checkpoints that already exist in stepped care and staging models. In stepped care, treatment begins with lower intensity ERP-based steps designed to reduce burden and improve access, then intensifies only when response is insufficient (Gilliam et al., 2010; Aspvall et al., 2021). For example, one adult stepped care protocol begins with a self-directed ERP phase with brief therapist support and steps up to twice weekly therapist delivered ERP when responder criteria are not met, with early step up permitted when symptoms or risk worsen (Gilliam et al., 2010). Another stepped care model begins with a brief, low intensity phase using ERP bibliotherapy with periodic clinician support and then moves to a standard course of therapist delivered ERP delivered at higher frequency for those who do not meet prespecified responder thresholds (Tolin et al., 2011). A pediatric stepped care trial retains the same architecture: an initial step is delivered first, then nonresponders at a prespecified follow up are offered additional face to face care (Aspvall et al., 2021). Across adult and pediatric protocols, escalation is triggered by defined nonresponse rather than by default intensity selection (Gilliam et al., 2010; Tolin et al., 2011; Aspvall et al., 2021).

Clinical staging adds phases of illness and prognosis. It distinguishes subthreshold, first episode, chronic, and refractory patterns using severity and course features once symptoms are identified and supports decision points that are not limited to early response alone. The staging framework also places explicit emphasis on prospective testing of lifestyle oriented interventions in earlier phases, which creates a legitimate place to evaluate metabolic interventions without turning this paper into a mechanisms review (Fontenelle and Yücel, 2019).

An adequate ERP trial is often operationalized as a structured course delivered over roughly 12 to 20 sessions, with outcome dependent on adherence to between session exposure practice and response prevention (Janardhan Reddy et al., 2017; Ojalehto et al., 2020; Tjelle et al., 2021). When symptom change remains below commonly used response thresholds and functional impairment remains clinically significant, the picture is best characterized as nonresponse rather than slow improvement, with operational definitions frequently using a Y-BOCS reduction below about 25 percent for nonresponse and 35 percent or greater for response (Veale et al., 2021; Mataix-Cols et al., 2022). At that point, guidance and ERP reviews describe increasing ERP intensity and considering higher levels of care when severity or treatment resistance warrants it, while also considering adjunctive or optimized treatments when response remains insufficient (Janardhan Reddy et al., 2017; Hezel and Simpson, 2019).

This moment of non-response after an adequate trial is central to the conceptual analysis developed here. One option is to escalate along the same axes that have already produced limited benefit, for example more frequent ERP sessions and higher SSRI doses. Another is to ask whether other modifiable factors, such as metabolic health, sleep, or inflammatory status, might be contributing to low readiness and whether targeting those factors could improve the person’s capacity to benefit from a second round of ERP. Stepped care and staging checkpoints are summarized as candidate testing windows for KMT (Table 2).

**Table 2:** Model anchored checkpoints for testing ketogenic metabolic therapy as an exposure and response prevention readiness adjunct.

Model and citation	Checkpoints defined in the model	Usual step-up action in the model	Where KMT may be introduced or studied
Gilliam et al. (2010)	Step 1 is a 6-week self-directed EX RP phase with minimal therapist guidance, nonresponse triggers step up, response is defined using clinically significant change on Y-BOCS.	Step 2 is therapist directed exposure delivered twice weekly.	At the Step 1 nonresponse checkpoint before Step 2 begins, and earlier when Step 1 cannot be completed because readiness is too low for between session exposure practice.
Tolin et al. (2011)	Step 1 is 6 weeks of low intensity counseling plus ERP bibliotherapy, nonresponders at 6 weeks step up to standard ERP.	Step 2 is therapist administered ERP delivered twice weekly across 17 sessions.	At the 6-week nonresponse checkpoint before Step 2 begins, and when Step 1 cannot be implemented because readiness barriers prevent sustained exposure practice.
Aspvall et al. (2021)	Step 1 is 16 weeks of internet delivered CBT, nonresponders at 3 month follow up are offered additional face to face CBT.	Additional face to face CBT is offered to nonresponders.	As a template for adult protocols, test KMT at a prespecified nonresponse checkpoint when readiness barriers prevent progression to the next care step.
Fontenelle and Yücel (2019)	Stage classification uses Y-BOCS ranges plus phase features, with subdivisions for first episode versus chronic course and for severe but potentially responsive versus severe and refractory illness.	Stage matched monitoring and escalation consistent with severity, phase, and complexity.	Test KMT in Stage I where risk features and low readiness justify prospective adjunct testing, and test KMT in chronic and severe but potentially responsive presentations where readiness remains low despite appropriate care progression.

Table summarizes stepped care and staging checkpoints that can define KMT testing windows and the primary endpoints to track. EX RP, Exposure response prevention; KMT, Ketogenic metabolic therapy; Y-BOCS, Yale Brown Obsessive-Compulsive Scale.

A clinically decisive question is whether ketogenic metabolic therapy increases the likelihood of completing an adequate next step of exposure and response prevention after a documented failure of the prior step. Two clinical windows align with these frameworks and keep the study question narrow. The first is when a defined ERP-based step has been delivered and response is insufficient on repeated symptom and functioning measures despite appropriate implementation. The second is when readiness remains too low to initiate or complete an adequate ERP dose because distress tolerance, cognitive capacity, or contextual feasibility does not support sustained between session exposure practice. In both windows, KMT is clinically meaningful only if it improves readiness and ERP engagement, defined by adherence to between session exposure practice and completion of an adequate ERP dose, alongside symptom severity and functioning tracked with repeated measures. This framing preserves ERP as the central intervention while giving stepped care a concrete way to test whether a metabolic adjunct restores capacity for exposure based treatment when standard sequencing has not (Gilliam et al., 2010; Tolin et al., 2011; Fontenelle and Yücel, 2019). Validated measures are available for ERP readiness and participation constructs, including alliance, treatment credibility and expectancy, between-session exposure and response prevention adherence, family accommodation, implementation acceptability and feasibility, and barriers to ERP initiation and continuation, including non-initiation and dropout (Horvath and Greenberg, 1989; Calvocoressi et al., 1999; Devilly and Borkovec, 2000; Mohr et al., 2010; Simpson et al., 2010; Clement et al., 2012; Weiner et al., 2017). Future trials of KMT as a readiness adjunct should prespecify instrument selection and timing so participation outcomes can be interpreted beyond retention counts (Montgomery et al., 2018). Research design and priority reporting recommendations relevant to evaluation of KMT as a candidate adjunct to ERP participation are provided (Table 3).

**Table 3:** Research priorities for KMT as a candidate adjunct to ERP participation in OCD.

Priority or decision point	Participation endpoints to report	Feasibility and acceptability indicators	Design and reporting priorities	Relevant categories of assessment	Research rationale and key sources
Entry assessment, initiating ERP and defining an adequate ERP trial.	ERP initiation rate, % referred attending ≥1 session, time from referral to 1st ERP session, early discontinuation timing, dropout before session 6, exposure progression milestone, hierarchy completed, response prevention plan documented, first between-session exposure assigned, prespecified adequate dose definition, protocol session range 12 to 20, plus prespecified criteria for receipt of ERP procedures.	Patient rated acceptability and tolerability of initiating exposure and response prevention work, brief structured items plus optional qualitative, reasons for non-initiation or early discontinuation including depression burden when present, secondary readiness domain, treatment credibility and expectancy assessed at baseline or early sessions as a decision relevant moderator, practical barriers preventing ERP initiation or continuation when present.	Prespecify adequate ERP target dose and procedural receipt criteria *a priori* and distinguish target dose from achieved dose. Define early discontinuation a priori, including session 6 as the early dropout cut point. For entry testing, prespecify KMT sequencing before or alongside early ERP when readiness constraints limit initiation or early continuity, then test whether change in prespecified readiness domains coincided with ERP initiation and exposure progression.	Initiation and early continuity, early dropout timing and reasons, readiness beliefs, credibility and expectancy, early exposure plan uptake and homework start.	OCD care gaps include under receipt of evidence-based psychotherapy and barriers to receiving ERP, supporting initiation and early discontinuation as primary delivery endpoints (Senter et al., 2021). Early dropout can be defined as discontinuation before session 6, supporting a prespecified early discontinuation cut point (Aderka et al., 2011). Typical ERP protocols use 12 to 20 sessions, supporting a prespecified adequate ERP trial target dose (Janardhan Reddy et al., 2017). Treatment credibility and expectancy can be measured before exposure treatment and predict exposure outcomes, supporting their use as a secondary readiness moderator at entry (Berg et al., 2025).
Adherence assessment, between session exposure practice and response prevention.	Between-session exposure completion, % assigned exposures attempted since last session, exposure quality, completion as assigned without compensatory rituals or safety behaviors, response prevention adherence, % urges resisted since last session and degree of ritual abstinence between sessions, session-by-session exposure phase adherence, hierarchy progress and homework review documented.	Patient reported tolerability of between-session exposure practice and response prevention, distress interference, avoidance, and ritual escalation during homework when present, fatigue and cognitive inefficiency constraints when present.	Define ERP dose by between-session exposure and response prevention, not attendance. Prespecify an adherence failure checkpoint and KMT sequencing at that checkpoint when adherence is insufficient for ERP procedures. Prespecify targeted readiness constraints, then test whether constraint change coincided with improved exposure and response prevention adherence and/or tolerance.	Exposure adherence, response prevention adherence, barriers to between-session practice tied to distress tolerance, fatigue, and cognitive efficiency when present.	ERP outcomes have been described as contingent on adherence to exposure and response prevention procedures outside sessions (Simpson et al., 2010; Hezel and Simpson, 2019). Because much of ERP practice occurs between sessions, adherence measures were developed to quantify follow-through with prescribed exposures and response prevention homework (Simpson et al., 2010; Ojalehto et al., 2020). Higher adherence has predicted lower posttreatment OCD severity (Hezel and Simpson, 2019; Ojalehto et al., 2020). These findings support specifying adherence as a primary endpoint in readiness adjunct designs.
Reassessment and step up decision, nonresponse or failure to achieve an adequate ERP trial.	Step completion is documented, with the prior ERP step completed as defined by the stepped-care protocol. Achieved dose and procedural receipt are reported using sessions received and documentation that exposure and response prevention are delivered as intended. Checkpoint response status is classified as nonresponse, partial response, or response using prespecified thresholds, with conservative response and remission classification when feasible. Step-up timing is recorded as scheduled reassessment versus earlier escalation when symptoms or risk worsen. Completion of the next step is documented, including intensified therapist-delivered ERP with adherence expectations specified.	Patient rated acceptability of escalation and reasons for declining or discontinuing step up care when present. Secondary readiness domain, credibility and expectancy assessed at checkpoint as a decision relevant moderator. Practical burden indicators, time, cost, and limited access to trained ERP clinicians when present.	KMT testing windows are anchored to documented nonresponse or failure to complete an adequate ERP trial, not to arbitrary timepoints. KMT sequencing is prespecified as pre-escalation to increase completion of the next ERP step after prior step failure, or concurrent to support adherence during intensified exposure demands. Response and remission definitions, and step-up triggers, are prespecified and aligned to stepped-care checkpoints and staging level when used.	Step completion and step-up outcomes. Response and remission classification. Secondary readiness beliefs, credibility and expectancy at checkpoint. Access constraints when present.	Stepped-care protocols begin with lower intensity interventions and step up when response is insufficient, supporting checkpoint-anchored adjunct testing rather than arbitrary timing (Gilliam et al., 2010; Tolin et al., 2011). Staging frameworks reinforce phase-matched checkpoint logic (Fontenelle and Yücel, 2019; Benatti et al., 2022). Conservative response and remission definitions reduce overestimation and strengthen escalation decisions (Mataix-Cols et al., 2022). Treatment credibility and expectancy can be assessed prior to exposure treatment and predict outcomes, supporting their use as secondary moderators at step-up decisions (Berg et al., 2025).
Clinical readiness assessment, depression, cognition, insight limiting ERP feasibility.	ERP hold and restart criteria when severe depression or suicidal ideation are present. Cognitive capacity markers, working memory and executive functioning indicators when feasible. Insight level tracked over time when relevant to readiness and response prevention. Functional impairment markers that constrain embedding exposure work into daily life.	Patient reported inability to plan, initiate, and sustain exposure tasks due to cognitive inefficiency or fatigue when present. High arousal, mood instability, or distress intolerance limiting ability to remain in exposures and sustain response prevention between sessions when present. Communication barriers preventing agreement on exposure goals and tasks when present.	Prespecify KMT as a readiness intervention before ERP reattempt when fatigue, cognitive inefficiency, limited insight, high arousal, or low distress tolerance limits exposure persistence and response prevention. Test whether change in prespecified readiness domains coincided with re-entry into exposure work and improved between-session adherence. Prespecify ERP hold, modification, and restart criteria, including holding ERP during severe depression and pausing full exposure work when distress intolerance prevents completing exposures without ritualizing. Measure working memory, executive function, insight, and baseline arousal or mood instability as moderators when feasible.	Mood burden and safety indicators when present. Arousal and distress intolerance indicators relevant to remaining in exposures and sustaining response prevention when present. Working memory and executive functioning indicators when feasible. Insight measures. Functional impairment indicators relevant to exposure feasibility.	ERP requires remaining in exposures while tolerating anxiety and sustaining response prevention, so high arousal and low distress tolerance can limit feasibility and should be assessed in readiness restoration studies (Janardhan Reddy et al., 2017). Guidelines note that in severe depression CBT and ERP may need to be held until recovery (Janardhan Reddy et al., 2017). Diminished working memory and communication factors predict poorer response to ERP-based CBT (Hamatani et al., 2020). OCD is associated with broad executive function impairments and depressive symptoms can contribute to cognitive deficits (Snyder et al., 2015; Mondal and Kumar, 2020). Insight can change over time and improvement in insight is associated with reduced OCD severity (Wolf et al., 2023).
Support context assessment, accommodation and access barriers shaping response prevention.	Family accommodation and proxy compulsions that undermine response prevention when present. Report exposure adherence and response prevention adherence within high-accommodation contexts when feasible. Care barriers when present, time and cost constraints, limited access to trained ERP clinicians.	Family and household willingness to reduce accommodation and align routines with response prevention plans when present. Environmental stability and interpersonal conflict indicators when relevant to adherence and continuation. Feasibility of integrating KMT routines within the home context when KMT is tested as an adjunct, added-demand burden.	Measure accommodation as a readiness context that can be targeted alongside response prevention plans. Document whether low readiness reflects access barriers rather than refusal. Prespecify whether accommodation and instability patterns that block ERP also moderate adherence and completion, then test this moderation.	Accommodation and support context indicators. Care barrier indicators when present. Adherence and completion outcomes stratified by psychosocial constraints when feasible.	Guidelines advise assessing family involvement, including accommodation and proxy compulsions, providing rationale for measuring accommodation as a contextual factor relevant to response prevention readiness (Janardhan Reddy et al., 2017). OCD has been described as undertreated, with limited use of ERP contributing to this gap, providing rationale for measuring access and delivery gaps (Senter et al., 2021). Stepped care studies have highlighted time, cost, and limited availability of trained ERP clinicians as constraints on ERP uptake, providing rationale for measuring practical barriers within readiness research (Tolin et al., 2011).
KMT implementation assessment, protocol fidelity, ketosis verification, monitoring aligned to ERP endpoints.	KMT initiation timing relative to the ERP checkpoint, entry, adherence failure, or nonresponse. Ketosis verification method and frequency, % monitoring completed. Diet adherence indicators and discontinuation timing. Alignment of KMT adherence with ERP adherence and step outcomes, between-session exposure practice, response prevention adherence, step completion.	Patient rated tolerability and monitoring burden. Support intensity, clinician follow-up, and adherence supports when present. AE reporting and reasons for discontinuation. Impact of KMT monitoring burden on ERP scheduling and between-session adherence when measured.	Specify KMT protocol, education, supplementation, monitoring plan, and discontinuation criteria. Prespecify ketosis verification targets and interpret adjunct effects cautiously when ketosis is unverified. Prespecify follow-up and laboratory monitoring aligned to protocol risk, and schedule KMT contacts to protect ERP access when delivered ERP dose and between-session adherence are primary endpoints.	Ketosis verification and monitoring completion. Laboratory safety monitoring and AE reporting. KMT adherence and discontinuation reasons. Integration of KMT fidelity measures with ERP adherence outcomes.	Consensus dietary therapy recommendations include regular follow-up, ketone monitoring, and periodic laboratory monitoring, supporting prespecified monitoring procedures in psychiatric KMT adjunct trials (Kossoff et al., 2018; Sethi et al., 2026). Clinical psychology guidance supports psychologist roles in implementation, adherence support, and multidisciplinary collaboration (Laurent et al., 2024). An OCD case report described KMT with ketone monitoring to support ERP, supporting alignment of KMT fidelity measures with ERP adherence and completion endpoints when testing KMT as a readiness adjunct (Laurent and Tague, 2025).

Research priorities for evaluating ketogenic metabolic therapy (KMT) as a candidate adjunct to exposure and response prevention (ERP) delivery in obsessive compulsive disorder (OCD). The table defines decision points for adjunct testing and specifies participation endpoints, initiation, between-session adherence, step completion, and step-up outcomes. It also specifies feasibility and acceptability indicators, contextual barriers, and KMT fidelity and monitoring elements needed for interpretability when participation is the primary evaluation target. Acronyms: AE, adverse event, CBT, cognitive behavioral therapy, ERP, exposure and response prevention, KMT, ketogenic metabolic therapy, OCD, obsessive compulsive disorder.

## 6 Conclusion

This conceptual analysis advances a readiness focused clinical framework for obsessive compulsive disorder that stays anchored to how ERP is delivered across stepped care. ERP effectiveness depends on whether the person can initiate, tolerate, and sustain repeated exposure. When readiness remains low after appropriate sequencing and escalation, the clinical problem is not a lack of ERP rationale, it is a persistent gap between what ERP requires and what the patient can reliably do.

Within this framing, KMT enters as a candidate adjunct that is clinically meaningful if shown it can improve readiness limiting capacities enough to make ERP feasible for patients. The contribution of this conceptual analysis is not a biological mechanism review of KMT as seen in the metabolic psychiatry literature. It is a clinician and research facing proposal that specifies where KMT can be tested, what must be measured, and what would count as success or failure from a psychological care standpoint. If it is concluded that KMT does not improve readiness and ERP engagement at defined decision points, it should not be carried forward in stepped care. However, if it does, it offers a concrete way to reopen exposure-based treatment for some patients.
